# Deep learning frameworks for protein–protein interaction prediction

**DOI:** 10.1016/j.csbj.2022.06.025

**Published:** 2022-06-15

**Authors:** Xiaotian Hu, Cong Feng, Tianyi Ling, Ming Chen

**Affiliations:** a Department of Bioinformatics, College of Life Sciences, Zhejiang University, Hangzhou 310058, China; b Department of Colorectal Surgery and Oncology, Key Laboratory of Cancer Prevention and Intervention, Ministry of Education, The Second Affiliated Hospital, Zhejiang University School of Medicine, Hangzhou, Zhejiang, China; c Cancer Center, Zhejiang University, Hangzhou, Zhejiang 310058, China; d The First Affiliated Hospital, Zhejiang University School of Medicine, Institute of Hematology, Zhejiang University, Hangzhou 310058, China

**Keywords:** Deep learning, Protein–protein interaction, Feature embedding, Biological prediction

## Abstract

Protein-protein interactions (PPIs) play key roles in a broad range of biological processes. The disorder of PPIs often causes various physical and mental diseases, which makes PPIs become the focus of the research on disease mechanism and clinical treatment. Since a large number of PPIs have been identified by *in vivo* and *in vitro* experimental techniques, the increasing scale of PPI data with the inherent complexity of interacting mechanisms has encouraged a growing use of computational methods to predict PPIs. Until recently, deep learning plays an increasingly important role in the machine learning field due to its remarkable non-linear transformation ability. In this article, we aim to present readers with a comprehensive introduction of deep learning in PPI prediction, including the diverse learning architectures, benchmarks and extended applications.

## 1 Introduction

The human genome codes about 500,000 diverse proteins and over 10,000 proteins can be produced throughout all time periods [1]. Most of the proteins operate in the form of complexes and about 130,000 to 650,000 different types of PPIs may occur in human body [2], [3], which are believed to be of terrific importance for almost all cellular processes. Moreover, a mass of non-covalent contacts between the side chains of amino acid residues take dominant responsibility for protein folding and interaction [4]. The cellular PPIs participate in almost all biological processes, ranging from metabolism, genetic pathways and signaling cascades, in which they serve for DNA replication and transcription, RNA translation, post-translational modifications, enzymatic reaction, energy generation, signal transduction, immunity and so forth. The massive information harbored in the protein interactions implies the functions and mechanisms of the associated pathways in cellular processes, and the clues to the therapies of human diseases. So important are these relationships among proteins that a vast number of *in vivo* and *in vitro* identification methods have been largely developed in the past decades. The *in vitro* methods include affinity chromatography, coimmunoprecipitation, nuclear magnetic resonance (NMR) spectroscopy, tandem affinity purification-mass spectroscopy (TAP-MS), X-ray crystallography, and protein microarrays [5]. As for *in vivo* methods, yeast two-hybrid, bimolecular fluorescent complementary (BiFC) and so forth have been widely utilized for PPI detection. Although the complex nature of PPI makes the *in vivo* and *in vitro* experiments time-consuming and labor-intensive, a large number of PPI data have been identified over decades. To date, more than one hundred related databases have been established and available online [6], like the Database of Interaction Proteins (DIP) [7], Search Tool for Retrieval of Interacting Genes/Proteins (STRING) [8], Biological General Repository for Interaction Datasets (BioGRID) [9], [10] and so forth.

**Fig. 1:**
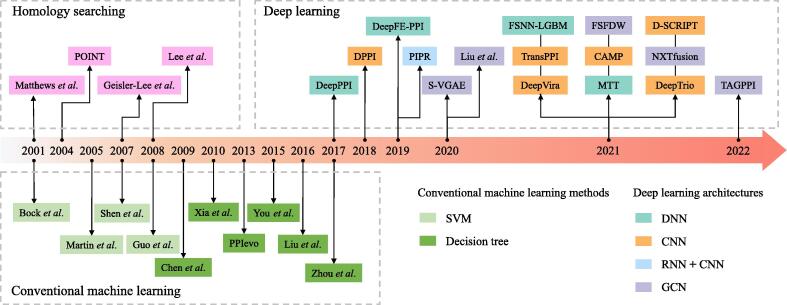
Timeline for computational PPI prediction methods.

The last decades have witnessed great progress in the field of computer science. With the fully sequenced genomes and proteomes, a number of innovative *in silico* methods for PPI identification have been proposed in the past decades. In the early stage, the computational methods mainly use the statistical characters and conserved patterns of proteins, as many functionally important proteins are conserved across species. The proteins sharing the homologous sequence patterns or structures may have a tendency of possessing the same interaction properties. Some of PPIs can be inferred by the homologous proteins across species [11]. Thereby, many approaches use ‘interologs’ (the conserved PPIs [12]) to predict PPIs among a diverse range of species [13], [14], [15], [16], and some of the predicted PPIs have been verified by further lab experiments. Later, the application of machine learning methods in PPI prediction can be traced back to 2001 [17]. The machine learning algorithms can be generally divided into three main categories: Supervised Learning (including Bayesian inference, decision tree, support vector machine (SVM), and artificial neural networks (ANNs)), Unsupervised Learning (like K-means and spectral clustering), and Reinforcement Learning. Among all of these machine learning methods, SVM aims to find an optimal hyperplane that separates the different labeled samples with a maximal margin. Many protein features, like conserved sequence patterns, 3D structures, domain compositions and corresponding gene expression can be leveraged by the SVM-based approaches [18], [19], [20], [21]. Decision tree-based methods recursively partition the sample space according to the diverse features of proteins. These features can be the primary sequences [22], [23], [24], [25], 3D structures [26] and domain composition [27], [28]. Some of the computational prediction methods and their timeline are shown in Fig. 1.

In the recent decades, ANNs (also known as deep learning) with the powerful non-linear transformation ability, have been drawing more and more attention and playing a more and more important role in a diverse range of fields. The deep learning-based approaches can achieve better performance compared with the conventional machine learning-based approaches in PPI prediction. Therefore, the scope of this article focuses on the protocol of deep learning for PPI prediction.

## 2 Preliminary

The primary goal of PPI prediction is to give a binary result that indicates whether a given pair of proteins interact or not. The performance of different approaches can be evaluated by a variety of metrics on the gold standard dataset.

### 2.1 Task definition

PPI prediction is usually a binary classification task. The objective of this task requires the deep learning models to learn a mapping function that takes as input various features of a given pair of proteins ($P_{1},P_{2}$), where $P_{1}$ and $P_{2}$ are two vectors in the same high-dimensional parameterized protein feature space, and outputs a prediction score in the range [0,1] indicating the probability of the protein interaction.

### 2.2 Databases

Different training and test data will lead to a variety of performance for approaches, so dataset selection is of vital importance. There are many databases that document a massive quantity of experimental PPI data, such as DIP [7], the Molecular INTeraction Database (MINT) [29], the Human Protein Reference Database (HPRD) [30], STRING [8], the Human Integrated Protein-Protein Interaction Reference (HIPPIE) [31], IntAct [32] and BioGRID [9]. *Saccharomyces cerevisiae* PPI data are widely used to train and evaluate the prediction methods [21], [33], [34], [35], [36]. The *S.cerevisiae* core dataset contains only the most reliable high-quality physical PPIs from DIP database. HIPPIE and HPRD are two widely used human PPI databases. DPPI [33] and Liu’s work [77] obtain the high confidence human PPI data by collecting the 10% top-scoring interactions from the HIPPIE database. DeepPPI [35] and DeepFE-PPI [36] use the HPRD database to build the human PPI dataset. Some of these PPI databases are shown in Table 1.

**Table 1:** Typical protein–protein interaction databases for deep learning models.^a^

Database	Proteins	Interactions	Organisms	URL	Confidence scores	Type of information	Used by
DIP [7]	28,850	81,923	*S. cerevisiae, E. coli, H. sapiens, A. thaliana and etc.*	https://dip.doe-mbi.ucla.edu/dip	Unavailable	Interactions	DeepPPI, DPPI, PIPR, DeepFE-PPI, Liu’s work, DeepTrio, FSFDW, TAGPPI, S-VGAE
HPRD [30], [72]	30,047	41,327	*H. sapiens*	https://hprd.org	Unavailable	Interactions, disease associations, domain annotations	DeepFE-PPI, DeepPPI, S-VGAE
HIPPIE [31], [73]	17,000	273,900	*H. sapiens*	https://cbdm.uni-mainz.de/hippie	Available	Interactions, disease associations	DPPI, Liu’s work,
BioGRID [9], [10]	82,082	1,244,672	*S. cerevisiae, R. norvegicus, H. sapiens, A. thaliana and etc.*	https://thebiogrid.org	Unavailable	Interactions, Go associations	DeepTrio, D-SCRIPT
STRING [8]	67,592,464	296,567,750	*S. cerevisiae, E. coli, H. sapiens, A. thaliana and etc.*	https://cn.string-db.org	Available	Interactions	PIPR, D-SCRIPT, MTT, TAGPPI
IntAct [32]	118,759	1,184,144	*S. cerevisiae, M. musculus, H. sapiens, A. thaliana and etc.*	https://www.ebi.ac.uk/intact	Available	Interactions	MTT
HPIDB [74]	16,332	69,787	Hosts and pathogens	https://hpidb.igbb.msstate.edu	Unavailable	Interactions, host and pathogen associations	DeepViral, TransPPI
MINT [29]	27,069	132,249	*S. cerevisiae, H. sapiens, M. musculus, R. norvegicus and etc.*	https://mint.bio.uniroma2.it	Available	Interactions	
RCSB PDB [75]	128,685	NA	*E. coli, H. sapiens, M. musculus, R. norvegicus and etc.*	https://www.rcsb.org	Unavailable	Complexes, structures, disease associations	CAMP, TransPPI

a NA, not available from the original paper.

The full protein sequences are usually retrieved from the Universal Protein Resource (UniProt) [37] database. To avoid the overestimation caused by the highly homologous sequences, a nonredundant subset is built by commonly removing the proteins with an identity threshold of 40% [33], [34], [35] using the CD-HIT [38], [39] software. Additionally, proteins with fewer than 50 amino acid residues are also removed in some studies [34], [35], [40].

### 2.3 Negative data construction

The negative dataset can be constructed by remolding the positive PPI data or directly collected from the non-interacting protein database like Negatome [41], [42]. The common method to construct the negative samples is to randomly pair the proteins in different sub-cellular locations and without observed evidence of interaction. The annotations of sub-cellular location on the proteins can be obtained from the Swiss-Prot [43] database. This negative data construction method is based on the expected sparsity of the protein interactome. Another negative data construction method is to shuffle the protein sequences [21], [40]. It has been proven that the possibility of the interaction can be deemed negligible if one sequence of a pair of interacting proteins is shuffled [44].

### 2.4 Evaluation criteria

There are six common evaluation metrics for model assessment, involving accuracy, precision, sensitivity, specificity, F1 score and Matthews correlation coefficient (MCC). Four indicators are used to calculate these metrics, including TP (true positive), TN (true negative), FP (false positive) and FN (false negative). These evaluation metrics are defined as follows:

(1) $$Accuracy=\frac{TP+TN}{TP+TN+FP+FN}$$

(2) $$Precision=\frac{TP}{TP+FP}$$

(3) $$Sensitivity=Recall=\frac{TP}{TP+FN}$$

(4) $$Specificity=\frac{TN}{TN+FP}$$

(5) $$F1\;score=\frac{2TP}{2TP+FP+FN}$$

(6) $$MCC=\frac{TP\times TN-FP\times FN}{\sqrt{\left(TP+FP\right)\left(TP+FN\right)\left(TN+FP\right)\left(TN+FN\right)}}$$

Two area-associated metrics are also used to evaluate the model performance. The receiver operating characteristic curve (ROC curve) illustrates the trend of the true positive rate against the false positive rate, and the area under it (AUROC) provides a comprehensive insight into the model discrimination ability for different samples. The precision-recall curve depicts the trend of recall against precision, and the area under the precision-recall curve (AUPR or AP) is useful when the test set contains an imbalanced number of positive and negative samples.

## 3 Deep learning methodology

**Fig. 2:**
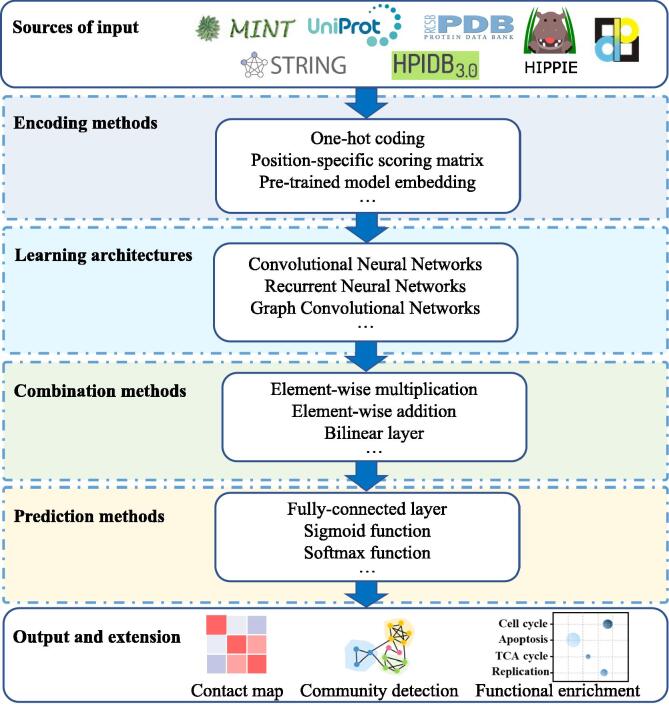
Overall deep learning framework for PPI prediction.

Generally, the Deep learning architecture can accept diverse types of input data for downstream analysis, such as primary sequence, domain component, protein 3D structure, network topology, gene expression, text mining, and so forth. Conventionally, protein 3D structure is considered to provide the most complete information for PPI prediction. Nevertheless, with the emergence of the intrinsically disordered proteins [45] and the induced fit theory [46], the primary sequences, as the most accessible information, become the main type of input for PPI computational identification. Besides, some network topology information, have been integrated into the sequence-based methods. The summary of the deep learning models for PPI prediction is shown in Fig. 2.

### 3.1 Encoding methods

As the computational methods take only the numerical data to train the models, it is an important phase to encode the proteins from the raw data. A number of sequence embedding methods have been developed to encode proteins. Different deep learning architectures require the input in different shapes. Generally, deep neural networks (DNNs) require a 1-D vector, while convolutional neural networks (CNNs) and other deep learning architectures require flexible input forms. They can be a 1-D vector for trainable amino acid lexicon embedding, a 2-D matrix derived from pre-trained models or the protein position-specific scoring matrix (PSSM) generated by Position-Specific Iterative (PSI)-BLAST.

#### 3.1.1 Artificially defined protein feature embedding

As a conventional protein encoding method, the handcrafted features extracted from protein sequences play an important role for converting symbolic information to the numerical vectors.

##### 3.1.1.1 DeepPPI

DeepPPI [35] uses a variety of statistical descriptors to characterize the structural and physicochemical natures of proteins, including amino acid composition, dipeptide composition, simplified attribute composition, transition and distribution. In addition, DeepPPI uses two higher-level descriptors to parameterize protein features. Quasi sequence order descriptor [47] describes the amino acid distribution patterns of specific physicochemical properties (Schneider-Wrede distance matrix [48] and Grantham chemical distance matrix [49]) along with the protein sequences. Another descriptor, amphiphilic pseudo-amino acid composition (APAAC) [50], also profiles the sequence-order information of the given proteins.

##### 3.1.1.2 S-VGAE

S-VGAE [51] chooses conjoint triad (CT) [20] as its encoding method. For CT encoding, all amino acids are classified into seven categories according to their electrical charges and side chain volumes. Next, a sliding window of size three counts the number of occurrences for each triad type with one step at a time. In this method, a protein can be encoded as:

(7) $$v=\left[n_{0},n_{1},{...,n}_{i},...,n_{q}\right]$$

where $n_{i}$ is the number of the $i_{\mathrm{th}}$ triad type and the length of v is 343 (7×7×7). This operator converts the raw protein sequence into the fixed-length vector for model input.

##### 3.1.1.3 FSNN-LGBM

In this method [52], pseudo amino acid composition (PseAAC) [53] and CT [20] descriptors have been employed to encode the protein sequences. PseAAC describes the correlation between residues in a certain distance, and CT clusters the amino acids based on the dipoles and volume of the residue side chains (the details of CT are described in Section 3.1.1.2).

#### 3.1.2 Evolutionary protein sequence embedding

The protein position-specific scoring matrix (PSSM) is usually leveraged in this method, which reveals the evolutionary profiles for the protein sequence in the form of the residue probability distributions in each position. PSSM is generated by applying Position-Specific Iterative (PSI)-BLAST searching in the protein database (like the UniRef50 database [54]). In DPPI [33] and TransPPI [55], the PSSM is a n×20 matrix S, where n is the length of the protein sequence and each element $s_{i,j}$ in the matrix denotes the probability of the $j_{\mathrm{th}}$ amino acid in the $i_{\mathrm{th}}$ position of the sequence. The only drawback of this method is that it needs an enormous effort for PSI-BLAST searching.

#### 3.1.3 Pre-trained model embedding

The existing PPI information (including experimentally verified interaction data, functional annotations, subcellular localizations, 3D structures and so forth) might lead to a limited training data that are not representative enough to ensure the robust, generalized and stable predictions of deep learning models. However, the pre-trained embedding models with a large number of priori knowledge can alleviate this problem to a certain extent.

##### 3.1.3.1 PIPR

PIPR [34] uses a property-aware amino acid lexicon to embed proteins, where the vectors describe the protein sequences from two aspects. The first part depicts the co-occurrence similarity of the amino acids, which is obtained by the pre-trained Skip-Gram model [56]. The Skip-Gram protein embeddings are optimized by minimizing the following loss function:

(8) $$l=-\frac{1}{\left|S\right|}\sum_{a_{t}\in S}\sum_{j=-c}^{c}p\left(a_{t+j}|a_{t}\right)$$

(9) $$p\left(a_{t+j}|a_{t}\right)=\frac{exp\left(a_{t+j}∙a_{t}\right)}{\sum_{k\notin U_{t}}exp\left(a_{k}∙a_{t}\right)}$$

where S denotes the set of all residues in the given protein, $a_{t+j}\in U_{t}$ is the neighboring residue of $a_{t}$, $U_{t}$ is the set of neighbor residues of $a_{t}$, which ranges from the (t-c) th residue to the (t+c) th residue, and c is the size of half context.

The second part depicts the similarity of electrostaticity and hydrophobicity among amino acids, where 20 amino acids are classified into 7 classes according to their dipoles and volumes of the side chains [20]. It is said that the amino acid lexicon can help PIPR better capture the contextual and physicochemical relatedness of amino acids.

##### 3.1.3.2 MTT

MTT [57] uses the UniRep model [58] to learn the representations of the corresponding proteins. The UniRep model is trained on the UniRef50 protein dataset (containing 24 million primary sequences) with the target of the next amino-acid prediction. The architectures of UniRep contain a 1,900-dimensional single-layer multiplicative long short-term-memory (LSTM) recurrent neural networks (RNNs) [59], a four-layer stacked multiplicative LSTM of 256 dimensions and a four-layer stacked multiplicative LSTM of 64 dimensions. The output of UniRep is a statistical representation containing the semantical, structural and evolutional information with 1900 dimensions [58].

##### 3.1.3.3 D-SCRIPT

D-SCRIPT [60] uses Bepler and Berger’s [61] pre-trained model which is a bidirectional LSTM (Bi-LSTM) trained on three different types of information. The primary task of this pre-trained model is to predict the global structural similarity between protein sequences as defined by the Structural Classification of Proteins (SCOP) database [62], which is a curated database of protein domain structures. Except for the global structural similarity, the pairwise residue contact maps for proteins and sequence alignment of similar proteins are both utilized for training the LSTM model. The embedding outputs from the Bepler and Berger’s model simultaneously present the local context and the global structure of the proteins.

##### 3.1.3.4 TAGPPI

TAGPPI [63] simultaneously leverages the sequence features and structural features to represent proteins. The structural features are learned by conducting graph convolution on the protein complex contact maps. The protein structure information is learnt by a spatial graph where the residues are the vertexes, and the contact map is the adjacency matrix. The amino acid representations in both sequence features and graph features are embedded by a pre-trained model SeqVec [64]. The SeqVec is obtained by training protein sequences on UniRef dataset with ELMo natural language processing model [65].

#### 3.1.4 Random walk-based protein feature embedding

In this encoding method, a semantic graph is first constructed by connecting different input entities. A number of synthetic sentences (which capture the co-occurrence of the input entities) are generated by the random walk algorithm. An embedding method (like Word2vec) is employed to learn a representation for each input entity from the synthetic sentences. The final embedding representations harbor the topological information among input entities.

##### 3.1.4.1 DeepFE-PPI

DeepFE-PPI [36] proposes a residue representation method named Res2vec (based on Word2vec [56]) to embed the input protein sequences. The Word2vec embedding method learns the semantic relations between the words in a corpus. In DeepFE-PPI, Word2vec is adapted to discover the co-occurrence information of residues in a protein database. The Res2Vec method maps the residue into a low-dimensional vector harboring the sequential and contextualized information.

##### 3.1.4.2 DeepViral

DeepViral [66] leverages the DL2Vec model [67] to embed protein ontology and phenotype information. The DL2Vec model first converts the protein features into a graph, and then the random walk method is employed to generate a corpus composed of a number of sentences capturing the topological information of the protein feature graph. The Word2Vec model is exploited to train the protein representations to capture their co-occurrence relations with other entities (including proteins, associated phenotypes and the Gene Ontology (GO) annotations) within the walks generated by DL2Vec.

#### 3.1.5 Trainable protein representation embedding

A trainable amino acid lexicon, which is initialized by a random 2-D matrix, is employed in this encoding method. Each row of the lexicon stands for an amino acid representation, whose weights can be updated in the backpropagation process. The protein representations are generated by retrieving the amino acid embeddings according to the indices provided by input sequences. NXTfusion [68] and DeepTrio [40] use this method to learn the protein representations for model input.

### 3.2 Learning architectures

The traditional neural network modules include the fully-connected layer, convolutional layer, recurrent layer and some structural tricks, like residual shortcut [69]. The fully-connected layer is usually employed to reshape the model variables . The convolutional layer is more inclined to learn the local features and analyze the associations between different regions, while the recurrent layer shows a propensity for preserving the contextualized and long-term ordering information. Recently, more and more graph learning methods, like Graph convolutional networks (GCNs), GraphSAGE [70] and Graph attention networks (GAT) [71], have been used for information aggregation, which combines the neighbor nodes’ features into the center node in the networks by mean pooling, summing, weighted averaging operations, or so forth. It is better for PPI prediction models to ensure a consistent prediction from arbitrarily ordered inputs(the featurization should be symmetric). Based on the above principle, the Siamese architecture [33], [34], [40] is usually employed, which contains two identical submodules sharing the same configuration and weights. In this section, we mainly describe the learning architectures adopted in the recently proposed deep learning methods for PPI prediction. All of these PPI prediction methods are listed in Table 2 and the reported performance is shown in Table 3.

**Table 2:** Recently proposed deep learning methods for PPI prediction.

Method	Year	Main learning structure	Sources of input feature	Encoding method	Combining method
DeepPPI [35]	2017	Multilayer Perceptron	Protein sequences	Seven sequence-based features (like amino acid composition)	Concatenation
DPPI [33]	2018	Convolutional Neural Networks	Protein sequences	Protein position specific scoring matrices (PSSM) derived by PSI-BLAST	Element-wise multiplication
DeepFE-PPI [36]	2019	Multilayer Perceptron	Protein sequences	Pre-trained model embedding (Word2vec [76])	Concatenation
PIPR [34]	2019	Bidirectional Gated Recurrent Unit and Convolutional Neural Networks	Protein sequences	Pre-trained model embedding (Skip-Gram [56]) and the similarity of electrostaticity and hydrophobicity among amino acids	Element-wise multiplication
S-VGAE [51]	2020	Graph Convolutional Neural Networks	Protein sequences and topology information of PPI networks	Conjoint triad (CT) method	Concatenation
Liu’s work [77]	2020	Graph Convolutional Neural Networks	Protein sequences and topology information of PPI networks	One-hot encoding	Concatenation
DeepViral [66]	2021	Word2Vec model and Convolutional Neural Networks	Protein sequences, phenotypes associated with human genes and pathogens, and the Gene Ontology annotations of human proteins	DL2Vec embedding model [67] and one hot encoding	Dot product
FSNN-LGBM [52]	2021	Multilayer Perceptron	Protein sequences	pseudo amino acid composition (PseAAC) and conjoint triad (CT) methods	Element-wise multiplication
TransPPI [55]	2021	Convolutional Neural Networks	Protein sequences	Protein position specific scoring matrices (PSSM) derived by PSI-BLAST	Concatenation
DeepTrio [40]	2021	Convolutional Neural Networks	Protein sequences	Trainable symbol lexicon embedding	Element-wise addition
FSFDW [78]	2021	Skip-Gram (Deepwalk)	Protein sequences and topology information of PPI networks	Sequence-based features selected by Louvain method and Term variance	Element-wise multiplication
NXTfusion [68]	2021	Multilayer Perceptron	Protein-Protein, Protein-Domain, Protein-Tissue and Protein-Disease relations	One-hot encoding	Bilinear transformation
MTT [57]	2021	Multilayer Perceptron	Protein sequences	Pre-trained model embedding (UniReo [58])	Element-wise multiplication
CAMP [79]	2021	Convolutional Neural Networks and Self-attention	Protein sequences, secondary structures, polarity, and hydropathy properties	Protein position specific scoring matrices (PSSM) calculated by PSI-BLAST and trainable symbol lexicon embedding	Concatenation
D-SCRIPT [60]	2021	Broadcast subtraction and multiplication, and Convolutional Neural Networks	Protein sequences	Pre-trained model embedding (Bepler and Berger’ work [61])	Broadcast subtraction and broadcast multiplication
TAGPPI [63]	2022	Convolutional Neural Networks and Graph attention networks	Protein sequences and structures	Pre-trained model embedding (SeqVec [64])	Concatenation

**Table 3:** The reported performance and efficiency of PPI deep learning methods.^a^

Method	Acc. (%)	Prec. (%)	Sen. (%)	Spec. (%)	F1 (%)	MCC (%)	AUC	AUPRC	Training time	Training environment	Benchmark
DeepPPI [35]	94.43	96.65	92.06	NA	NA	88.97	NA	NA	369 s	Intel Xeon E2520 CPU with 16G memory	*S. cerevisiae* Core Subset from DIP
DPPI [33]	94.55	96.68	92.24	NA	NA	NA	NA	NA	NA	32 AMD 6272 CPUs	*S. cerevisiae* core subset from DIP
DeepFE-PPI [36]	94.78	96.45	92.99	NA	NA	89.62	NA	NA	1008 s	Intel Core i5-7400 with 16G memory	*S. cerevisiae* core subset from DIP
PIPR [34]	97.09	97.00	97.17	97.00	97.09	94.17	NA	NA	150 s	NVIDIA GeForce GTX 1080 Ti GPU	*S. cerevisiae* core subset from DIP
S-VGAE [51]	99.15	98.90	99.41	98.89	99.15	NA	NA	NA	NA	NVIDIA GeForce GTX 1080 GPU with 7 GB memory	*H. sapiens* PPIs from HPRD
Liu’s work [77]	95.33	97.02	93.55	NA	NA	NA	NA	NA	NA	NA	*S. cerevisiae* core subset from DIP
DeepViral [66]	NA	NA	NA	NA	NA	NA	0.800	NA	NA	Nvidia Tesla V100 GPU	Host and pathogen PPIs from HPIDB
FSNN-LGBM [52]	98.70	99.11	98.28	99.12	NA	97.41	0.997	NA	NA	NA	*S. cerevisiae* core subset from DIP
DeepTrio [40]	97.55	98.95	96.12	98.98	97.52	95.15	NA	NA	NA	NVIDIA Tesla P100 GPU with 16 GB memory	*S. cerevisiae* PPIs from BioGRID
FSFDW [78]	NA	NA	NA	NA	NA	NA	0.794	NA	NA	NA	*E. coli* PPI dataset
NXTfusion [68]	NA	NA	NA	NA	NA	NA	0.988	0.778	NA	NA	*H. sapiens* PPIs used in FPClass [93]
MTT [57]	NA	93.53	94.05	NA	93.79	NA	0.980	0.980	NA	NVIDIA GTX 1080-Ti GPU with 11 GB memory	VirusMINT database
CAMP [79]	NA	NA	NA	NA	NA	NA	0.872	0.641	2 h	48 CPU cores and one NVIDIA GeForce GTX 1080Ti GPU	Protein-peptides interactions from the RCSB PDB and DrugBank
D-SCRIPT [60]	NA	72.8	27.8	NA	NA	NA	0.833	0.516	3 days	A single 32 GB GPU	*H. sapiens* PPIs from STRING
TAGPPI [63]	97.81	98.10	98.26	98.10	97.80	95.63	0.977	NA	NA	NVIDIA TITAN RTX with 24 GB memory	*S. cerevisiae* PPIs from DIP

a NA, not available from the original paper.

#### 3.2.1 Fully-connected based learning architectures

##### 3.2.1.1 DeepPPI

A variety of mathematical descriptors have been leveraged in DeepPPI [35] to extract the structural and physicochemical properties of protein sequences. The encoded vectors from two input proteins are separately passed through four stacked fully-connected layers and concatenated in the merging layer. The output of DeepPPI is a binary vector indicating whether the given protein pair interacts or not. More precisely, “1,0” denotes no interaction, whereas “0,1” stands for interaction.

##### 3.2.1.2 DeepFE-PPI

The learning framework of DeepFE-PPI [36] contains two separate DNN modules. Each of them possesses four stacked fully connected layers, which capture the high-level features hidden in the input vectors. In the prediction phase, the resulting outputs of DNN modules are firstly concatenated and then analyzed by two fully connected layers. Some widely used tricks like batch-normalization layers and dropout layers are attached to each fully connected layer except for the final output layer.

##### 3.2.1.3 FSNN-LGBM

After encoding the protein sequences, the feature vectors are artificially expended using the functional expansion method, which is proposed and described in [80]:

(10) $$\emptyset \left(D_{i}\right)=\left\{\begin{array}{c} D_{i}\left(1\right),\sin\Pi \left(D_{i}\left(1\right)\right),\cos\Pi \left(D_{i}\left(1\right)\right),\sin2\Pi \left(D_{i}\left(1\right)\right)\cdots ,\cos k\Pi \left(D_{i}\left(1\right)\right)\cdots \\ D_{i}\left(n\right),\sin\Pi \left(D_{i}\left(n\right)\right),\cos\Pi \left(D_{i}\left(n\right)\right),\sin2\Pi \left(D_{i}\left(n\right)\right)\cdots ,\cos k\Pi \left(D_{i}\left(n\right)\right) \end{array}\right)$$

where $\emptyset \left(D_{i}\left(n\right)\right)$ stands for the functional expansion of $n_{\mathrm{th}}$ attribute of $i_{\mathrm{th}\;}$ input unit in dataset D, and $\emptyset \left(∙\right)$ is the mathematical function, like sine and cosine.

Each element in the expanded input is sent to a fully connected layer, and integrated by element-wise summation for one protein representation. The integrated features of two input proteins are combined by an element-wise multiplication after they are passed through afully connected layer, and generate a 128-dimensional feature vector. The abstraction features are subsequently rescaled using min–max normalization.

As a hybrid model, the light gradient boosting machine (LSBM) [81] is incorporated into the FSNN-LGBM model for giving a more accurate probability of PPI.

##### 3.2.1.4 MTT

After protein feature encoding, the protein embeddings are passed through one hidden fully-connected layer with Rectified Linear Unit (ReLU) activation to extract the latent features. The two resulting representations derived from the fully-connected layer are firstly combined with an element-wise product, and then passed through a linear layer followed by the Sigmoid activation for PPI prediction.

#### 3.2.2 Convolution based learning architectures

##### 3.2.2.1 DPPI

DPPI [33] mainly uses the convolutional module to extract and analyze the underlying features of proteins as the following objective function:

(11) $$h=Pool\left(ReLU\left(Batch\left(conv\left(S\right)\right)\right)\right)$$

where S and h are the input vector and the output vector of the convolutional module, respectively. Meanwhile, DPPI employs the random projection module for enabling the model to distinguish the homodimeric and heterodimeric interactions, which projects the learned protein representations into a subspace using a pair of pseudo-orthogonal random weight vectors as follows:

(12) $$R_{1}=ReLU\left(Batch\left(\left[W^{1}\left|\right|W^{2}\right]h_{1}\right)\right)$$

(13) $$R_{2}=ReLU\left(Batch\left(\left[W^{2}\left|\right|W^{1}\right]h_{2}\right)\right)$$

where $W^{1}$ and $W^{2}$ are two projection matrices, || denotes the concatenation operation, and $R_{1}$ and $R_{2}$ are two outputs of the random projection module.

In the prediction phase, DPPI uses element-wise multiplication to combine the information of the given pairs of proteins. A linear layer followed with the Sigmoid layer transforms the combined vector into an output score, which indicts the probability of PPI. The model is optimized by the following loss function:

(14) $$\begin{array}{c} l\left(\hat{y},y\right)=\ln\left(1+\exp\left(-y\hat{y}\right)\right) \end{array}$$

where $\hat{y}$ is the output score before the Sigmoid layer,y is the true label of the given pair of proteins, and y=1 if there is an interaction, or 0 otherwise.

##### 3.2.2.2 DeepViral

DeepViral [66] extracts protein features from two individual components. A phenotype model captures the GO annotation and associated phenotype information with a fully-connected layer. Another model extracts the latent information from the amino acid sequences of the human and virus proteins, which contains a convolutional layer and a fully-connected layer. These two aspects of feature vectors are concatenated into a joint representation for the human protein and the virus protein, respectively. A dot product, along with the Sigmoid activation function, is performed over the two protein representations (human and virus) to compute the probability of human and virus protein interaction.

##### 3.2.2.3 TransPPI

This approach [55] employs four connected convolutional layers followed with the pooling layers within a Siamese-like architecture to capture the latent patterns in the input protein sequence. The prediction module concatenates a pair of protein representations generated from two identical sub-networks and passes them through three stacked fully-connected layers followed with the leakyReLU activation. The final probability value for interaction is defined by the Softmax activation function.

##### 3.2.2.4 DeepTrio

DeepTrio [40] employs multiple parallel convolutional learning architecture to perform binary PPI prediction. The query protein sequences are embedded by a learnable amino acid lexicon. Before the feature extraction module, the embedding vectors will firstly be masked according to different preprocessing strategies. By masking the whole sequence of one protein in each training case, the ‘single-protein’ data have been constructed and the model outputs the final vectors that contain three elements indicating the probabilities of interaction, non-interaction and single-protein. In addition, DeepTrio is extended to illustrate the effect of each residue in a protein on PPI.

##### 3.2.2.5 CAMP

CAMP [79] integrates multifaceted features, including the protein primary sequences, second structures, physicochemical properties and protein evolutionary information, to construct the input protein profiles. These feature vectors are concatenated together after the trainable embedding layers or fully-connected layers, and then the outputs are passed through three connected convolutional layers and a global max pooling layer to unify and extract the hidden contextual features. CAMP additionally adopts the self-attention layer to learn the long-dependencies between residues in protein sequences. CAMP concatenates the convolution outputs and the self-attention outputs to construct the resulting protein profiles. Finally, CAMP uses three fully-connected layers to extract latent features from the combined vectors and predicts whether the given pairs of proteins interact.

##### 3.2.2.6 D-SCRIPT

D-SCRIPT [60] uses a pre-trained Bi-LSTM model to generate the structurally informative representations of proteins. These protein embeddings are firstly projected into a lower-dimensional vector for the downstream analysis. The low-dimensional embeddings are used to calculated the protein contact map by broadcast subtraction and broadcast multiplication operations. The contact map denotes the locations of residue contacts between protein structures. In the prediction phase, the contact map is summarized into a single score that indicates the probability of interaction.

#### 3.2.3 Recurrent based learning architecture

##### 3.2.3.1 PIPR

PIPR [34] assembles convolution layers [82] and residual gated recurrent units (GRU) [83] as a residual recurrent convolutional neural network (RCNN) encoder to represent the proteins, which can effectively capture the local features and the long-term ordering information of the sequences. The residual shortcut [69], which adds the identity mapping of the GRU inputs to their outputs, prevents the model from the vanishing gradient problem and improves the learning abilities of the neural layers [84]. After the encoder, two protein vectors are combined using element-wise multiplication. In addition, PIPR is extended to a more generalized application scenarios for interaction type prediction and binding affinity estimation, by adjusting the training set and the training targets of the deep learning model.

#### 3.2.4 Graph learning-based architectures

##### 3.2.4.1 S-VGAE

S-VGAE [51] uses a variational graph auto-encoder [85] to learn the latent features of proteins. The encoder of the variational graph auto-encoders (VGAE) uses the GCNs to learn the mean values μ and standard deviation values σ of the gaussian distribution for the input nodes from the protein network graph and feature matrix. The encoder projects the initial coding of sequences into a low-dimensional embedding z. The decoder computes the inner product of a pair of protein embeddings $z_{i}$ and $z_{j}$ to reconstruct an approximation of the actual adjacency matrix, which is used to calculate the loss of the model. Specially, S-VGAE assigns different weights to the adjacency matrix, since different network edges have different confidence and different impacts on the graph learning. Finally, S-VGAE sends the concatenation of $z_{i}$ and $z_{j}$ through multiply fully-connected layers followed by ReLU activation to output a binary vector indicating whether there exists an interaction between the given pair of proteins.

##### 3.2.4.2 Liu’s work

This approach, proposed by Liu et al. [77], integrates the protein sequences and network information to identify PPIs. In the encoding phase, the proteins are represented by integrating the sequence information and the topology information in the network. The protein sequence information is represented using one-hot encoding method, where each amino acid in the given sequence is encoded as a 20-dimensional vector. The topology informationis represented wit the position and relation information in PPI networks of the given protein. Each node in the graph is initially set as a one-hot encoding vector, whose length is the number of proteins in the network. To capture the topology information of a given protein in the PPI networks, GCNs has been leveraged to aggregate the information from neighbor nodes, which is described as below:

(15) $$h_{i}^{\left(l+1\right)}=\sigma \left(\sum_{j\in N_{i}}\frac{1}{c_{\mathrm{ij}}}h_{j}^{\left(l\right)}W^{\left(l\right)}\right)$$

where $h_{i}$ is the hidden representation of protein i, $N_{i}$ is the set of the neighbors of protein i, $c_{\mathrm{ij}}$ is a normalization constant of the edge between protein i and protein j, W is the layer-specific weight, and $\sigma \left(∙\right)$ is a non-linear activation function.

The protein sequence information and topology information are concatenated to get the final protein representation. In the prediction phase, each protein of an input pair is passed through four fully connected layers to extract the high-level features. In addition, to avoid over-fitting and make the loss convergence faster, batch normalization and dropout have been leveraged.

##### 3.2.4.3 FSFDW

FSFDW [78] uses a Deepwalk-based method to embed the protein nodes. The initial features of proteins are divided into a group of clusters using the Louvain [86] algorithm. Next, the optimal features from each cluster are collected with the term variance criterion. FSFDW learns the topological information of the protein nodes by the Deepwalk method [87] that generates the fictitious protein sentences for downstream analysis. FSFDW uses a word2vec method, Skip-Gram [76], to take as input these sentences and learn the semantical similarity of input proteins. To address a major drawback of the Deepwalk method that treats every node in the network equally, FSFDW uses the structural similarity and the feature-based similarity to calculate the weights of the edges between node pairs. After the Skip-gram model, two protein vectors are combined by the Hadamard operator and then fed into the classifier for link prediction.

##### 3.2.4.4 NXTfusion

Relation graph factorization with the deep learning framework has been recently used for performing inference over a wide range of tasks in multiple scenarios and shows a good performance in biological entity relation prediction [68], [88]. NXTfusion [68] extends the conventional matrix factorization paradigm to making inference over multiple entity-relation (ER) graphs based on neural networks. Since NXTfusion can adopt arbitrary ER graphs, a heterogeneous range of additional features have been attached to the main binary PPI network graph, which are the Protein-Domain, Protein-Disease and Protein-Tissue graphs. NXTfusion is optimized by minimizing the following objective function:

(16) $$\underset{W,e}{\mathrm{argmin}}\sum_{R_{i,j}\in R}\omega_{i,j}L_{i,j}\left(R_{i,j},M_{i,j}\left(f_{i}\left(e_{i}\right),f_{j}\left(e_{j}\right)\right)\right)$$

where W are the trainable weights of the neural networks, $e_{i}$ are the embedding of the input entity, $f_{i}$ is the feed-forward layer, $M_{i,j}$ is the bilinear layer, $R_{i,j}$ is the observed relation between a pair of entities, and $\omega_{i,j}$ is the relation-specific scale factor.

The additional ER graph learning will also update the protein entity representations. Accordingly, the resulting protein representations involve the information from not only Protein-Protein graph, but also Protein-Domain, Protein-Disease and Protein-Tissue graphs, which improves the model generalization ability and prediction performance.

##### 3.2.4.5 TAGPPI

The embedding module of TAGPPI [63] produces two types of protein profiles including the sequence and spatial information. The sequence features are computed with three stacked one-dimensional convolution layers. The spatial graph information is extracted by GAT. The two types of protein feature vectors are fused into one vector with a weighted addition operator. After obtaining the pairwise protein representations, they are concatenated and fed into multiply fully-connected layers followed with ReLU activation to predict the probabilities of interaction.

### 3.3 Combining methods

Since the model needs to use the pairwise inputs to predict the probability, it is an essential phase to combine two representations of proteins into one vector for subsequent analysis. Diverse methods have been employed to conduct the combination operation. The element-wise multiplication is a commonly used method to combine two vectors [33], [34] while conserving the symmetric relations of the input proteins. In addition, element-wise addition [40], concatenation [35] and bilinear transformation [68] are also used to perform the combination operations.

### 3.4 Output and extensions

The resulting outputs of PPI prediction usually denote the probability of interactions, which are usually generated from Sigmoid layer or softmax layer. With the predicted PPIs, several extensive functions are developed for investigating the residue importance, detecting the protein function, and so forth.

#### 3.4.1 Important residue detection and visualization

Due to lack of interpretability, deep neural networks have been viewed as ‘black box’ and cannot give the distinctive features for each class. Recently, several visualization techniques for the deep learning method have been developed in biological field, like DeepBind [89], DeepSig [90] and DeepChrome [91]. Also, a few visualization methods have been leveraged in the PPI field. DeepTrio [40] provides an intuitive protein portrait by masking each amino acid of a protein and calculating its contribution to the prediction. D-SCRIPT [60] constructs an inter-protein contact map by performing broadcast subtraction and multiplication on two protein embeddings. The contact map is optimized to be a sparse matrix with a relatively small number of high-probability interaction regions by minimizing its magnitude loss.

#### 3.4.2 Functional module inference

D-SCRIPT [60] uses spectral clustering to perform the functional module detection in the predicted PPI networks, and generates 384 functional modules annotated by GO terms from FlyBase [92]. These predicted functional clusters harbor a relatively high average within-cluster similarity, which shows that D-SCRIPT have learned the accurate functional characterizations of the proteins during the training process.

## 4 Discussion

The advancement of the deep learning algorithm boosts the development of biological prediction *in silico* in the past decades, which severs as a starting point for further lab verification. The accumulation of more and more identified PPIs along with their primary sequences provides substantial training data for the computational models. Thus, an increasing number of sequence-based approaches have been developed to identify PPIs*.* As it is shown in Table 3, *S. cerevisiae* core subset from DIP has become the most commonly used benchmarks among a variety of datasets. Besides, some additional features beyond the primary sequences, like domain composition, secondary structures and 3D structures, improve the performance of the models. With the progress of the deep learning algorithms, the paradigm of PPI prediction has also developed. Multilayer Perceptron (MLP) shows increased performance for PPI prediction compared to the traditional machine learning methods in the initial stage of deep learning development. However, its learning structure limits the flexibility of the model input. Subsequently, CNNs effectively downsize the number of parameters by sharing convolutional window weights and learning the local features of inputs. Furtherly, RNNs can better capture the contextualized and long-term ordering information from the sequences. Specially, the combination of CNNs and RNNs along with residual shortcut tricks (RCNN architecture) achieves excellent and robust performance in PPI prediction [34]. Recently, the graph learning models provide a new insight into the non-Euclidean domain knowledge and show a powerful ability to construct dependencies and comprehend global characteristics of the network data. The graph neural networks may make the model learn the complex relationships among protein interaction networks better. Moreover, some downstream analyses, like visualization and functional module detection, make the models more interpretable. For example, DeepTrio uses a masking method to calculate the importance of each amino acid residue and D-SCRIPT constructs the inter-protein contact map by performing broadcast subtraction and multiplication on two protein representations. However, a number of other visualization techniques are expected to be leveraged in PPI prediction, like the network-centric approach and the deep Taylor decomposition approach, which may render a better visual presentation. With the help of deep learning methods, genome-scale PPI networks can also be reconstructed *in silico*, and protein functional modules can be inferred through network mining.

Although the deep learning framework shows a superior performance in the PPI prediction task, there are still some problems that need to be addressed. The aforementioned deep learning methods consider the PPI prediction as a binary classification task. However, in the real biological process, the protein complex may be composed of three or more component proteins, and only two of them cannot interact and form a stable complex. Therefore, a strategy that considers the comprehensive protein interaction information is important for the PPI prediction. Recently, some useful explorations have been made in this direction. TADW-SC [88] uses k-means clustering algorithm to reconstruct the PPI network and uses a community detection method for finding the protein complexes sharing the higher edge density and homogeneous features. Furthermore, the reliability of the datasets can also affect the prediction performance of deep learning models. False positives may still exist even though all the PPIs are validated by two independent experiments. In addition, the PPI prediction models also lack the sufficient negative PPI cases for training, although the negative samples can be constructed by randomly pairing the proteins in different sub-cellular fractions. For reducing the randomness, a large number of negative samples should be constructed, while it will also lead to the extremely imbalanced data distribution .

## CRediT authorship contribution statement

**Xiaotian Hu:** Conceptualization, Writing – original draft, Writing – review & editing, Visualization. **Cong Feng:** Writing – original draft, Writing – review & editing, Visualization. **Tianyi Ling:** Writing – review & editing. **Ming Chen:** Conceptualization, Supervision, Resources, Project administration, Funding acquisition, Writing – review & editing.

## Declaration of Competing Interest

The authors declare that they have no known competing financial interests or personal relationships that could have appeared to influence the work reported in this paper.
